# NMDAR2B tyrosine phosphorylation regulates anxiety-like behavior and CRF expression in the amygdala

**DOI:** 10.1186/1756-6606-3-37

**Published:** 2010-11-30

**Authors:** Mina Delawary, Tohru Tezuka, Yuji Kiyama, Kazumasa Yokoyama, Takeshi Inoue, Satoko Hattori, Ryota Hashimoto, Hisashi Umemori, Toshiya Manabe, Tadashi Yamamoto, Takanobu Nakazawa

**Affiliations:** 1 Division of Oncology, Institute of Medical Science, University of Tokyo, 4-6-1 Shirokanedai, Minato-ku, Tokyo 108-8639, Japan; 2 Division of Neuronal Network, Institute of Medical Science, University of Tokyo, 4-6-1 Shirokanedai, Minato-ku, Tokyo 108-8639, Japan; 3 Division of Systems Medical Science, Institute for Comprehensive Medical Science, Fujita Health University, 1-98 Dengakugakubo, Kutsukake-cho, Toyoake, Aichi 470-1192, Japan; 4 Core Research for Evolutional Science and Technology (CREST), Japan Science and Technology Agency (JST), 4-1-8 Hon-cho, Kawaguchi, 332-0012, Japan; 5 Molecular Research Center for Children's Mental Development, United Graduate School of Child Development, Osaka University, Kanazawa University, and Hamamatsu University School of Medicine, Yamada-Oka, Suita, Osaka 565-0871, Japan; 6 Department of Psychiatry, Osaka University Graduate School of Medicine, Yamada-Oka, Suita, Osaka 565-0871, Japan; 7 Molecular & Behavioral Neuroscience Institute and Department of Biological Chemistry, University of Michigan Medical School, Ann Arbor, MI 48109-2200, USA; 8 Division of Genetics, Institute of Medical Science, University of Tokyo, Tokyo 108-8639, Japan

## Abstract

**Background:**

Anxiety disorders are a highly prevalent and disabling class of psychiatric disorders. There is growing evidence implicating the glutamate system in the pathophysiology and treatment of anxiety disorders, though the molecular mechanism by which the glutamate system regulates anxiety-like behavior remains unclear.

**Results:**

In this study, we provide evidence suggesting that tyrosine phosphorylation of the NMDA receptor, an ionotropic glutamate receptor, contributes to anxiety-like behavior. The GluN2B subunit of the NMDA receptor is tyrosine-phosphorylated: Tyr-1472 is the major phosphorylation site. Homozygous knock-in mice that express a Tyr-1472-Phe mutant of GluN2B, which prevents phosphorylation of this site, show enhanced anxiety-like behavior in the elevated plus-maze test. Expression of corticotropin-releasing factor (CRF), which is important for the regulation of anxiety-like behavior, is increased in the amygdala of the knock-in mice. Furthermore, injection of CRF receptor antagonist attenuated the enhanced anxiety-like behavior of the knock-in mice. We also show that elevated plus-maze exposure simultaneously induced de-phosphorylation of Tyr-1472 and increased CRF expression.

**Conclusions:**

These data suggest that Tyr-1472 phosphorylation on GluN2B is important for anxiety-like behavior by negative regulation of CRF expression in the amygdala.

## Background

Anxiety is commonly experienced and typically adaptive; however, excessive and dysfunctional anxiety leads to serious disorders. Anxiety disorders are the most prevalent class of psychiatric disorders in many countries [1]. Compounds that target of γ-aminobutyric acid and the serotonergic systems have received great attention within the development of treatments for anxiety disorders [2]. As some forms of anxiety are relatively resistant to treatment with these compounds, which include benzodiazepines and selective serotonin reuptake inhibitors, it has become increasingly apparent that alternative treatment strategies are needed. Recently, the glutamatergic system, the major mediator of excitatory synaptic transmission in the mammalian brain, has been the focus of pathophysiological studies of human anxiety disorders [3]. In rodents, *N*-methyl-D-aspartate (NMDA) receptor antagonists show anxiolytic effects in several test scenarios including the elevated plus-maze test [4,5]. While these reports point to the involvement of NMDA receptor-mediated signaling in the regulation of anxiety-like behaviors, molecular dissection of the role of NMDA receptor-mediated signaling is difficult because glutamate exerts its effects on various neural functions in a highly complex manner [6].

The NMDA receptor is crucial for neural development, synaptic plasticity, neuronal excitotoxicity, and behavior [6-9]. The NMDA receptor is composed of the GluN1 and GluN2 subunits: the GluN1 subunit is essential for the function of NMDAR channels, whereas the GluN2 subunits (GluN2A, GluN2B, GluN2C, and GluN2D) determine the characteristics of NMDAR channels by forming different heteromeric configurations with the GluN1 subunit [6]. The function of NMDA receptor-mediated signaling is in part regulated by Src tyrosine kinase-mediated phosphorylation of the GluN2 subunit [10,11]. Previous studies have found that Tyr-1325 and Tyr-1472 are the principal tyrosine phosphorylation sites on the GluN2A and the GluN2B subunits, respectively [12,13]. Genetically engineered mice expressing the Y1325F mutation of GluN2A show antidepressant-like behavior, but their other neural functions, such as hippocampal-dependent learning, are normal [12]. Alternatively, mice expressing the Y1472F mutation of GluN2B show a selective impairment in amygdala-dependent fear-learning [13]. Considering the versatile role of the NMDA receptor in various neural functions [6], the phenotypes of these mutant mice are milder than expected: thus these mice provide valuable models in which to dissect the molecular basis of specific behaviors including anxiety-like behavior.

Corticotropin-releasing factor (CRF), which is highly abundant in the amygdala as well as in the paraventricular nucleus of the hypothalamus, plays an important role in regulating anxiety-like behavior [14]. Patients suffering from anxiety disorders often have increased CRF levels in their cerebrospinal fluid [15,16]. In rodents, intracerebro-ventricular delivery of CRF is anxiogenic [17]. Likewise, transgenic mice overexpressing CRF exhibit increased anxiety-like behavior [18]. Conversely, CRF_1 _receptor knockout mice have reduced anxiety [17]. Injection of CRF antagonists or CRF_1 _receptor antisense oligonucleotide into the amygdala reduces stress-induced anxiety-like behavior [19,20]. These results collectively show that CRF plays a key role in the regulation of anxiety-like behavior particularly in the amygdala. Therefore understanding the molecular mechanism of the regulation of CRF expression in the amygdala is important.

In the present study, using behavioral, pharmacological, and biochemical approaches with knock-in mice in which the Tyr-1472 of GluN2B is mutated to phenylalanine (GluN2B-YF), we have identified Tyr-1472 phosphorylation as a regulator of CRF mRNA expression and anxiety-like behavior.

## Results

### Enhanced anxiety-like behavior of GluN2B-YF mice

**Figure 1:**
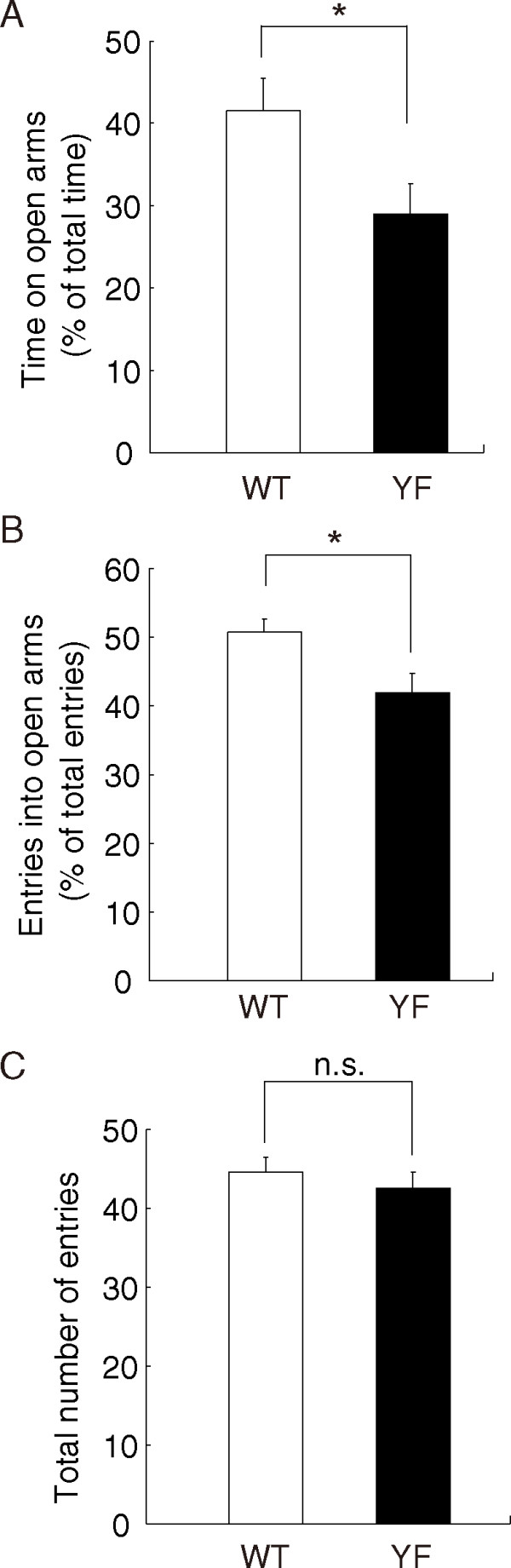
**Enhanced anxiety-like behavior of GluN2B-YF mice in the elevated plus-maze test**. **(A)**, **(B) **GluN2B-YF mice spent less time exploring the open arms (WT, *n *= 28; YF, *n *= 31; F_(1,57) _= 5.516, *p *< 0.05, one-way ANOVA) (A) and made fewer entries into the open arms (WT, *n *= 28; YF, *n *= 31; F_(1,57) _= 6.908, *p *< 0.05, one-way ANOVA) (B) during the elevated plus-maze test. **(C) **Total number of entries of GluN2B-YF mice into the open and closed arms was not significantly different from that of WT mice (WT, *n *= 28; YF, *n *= 31; F_(1,57) _= 0.490, *p *> 0.4, one-way ANOVA). The asterisk indicates significant genotype differences. n.s., not significant.

Given that we previously found that GluN2B-YF mice show a selective impairment in amygdala-dependent learning [13], we evaluated amygdala-dependent anxiety-like behavior in GluN2B-YF mice using the elevated plus-maze (EPM) test, one of the most popular behavioral tests for research on anxiety [21]. The measures of anxiety are the percentage of time spent in the open arms and the percentage of open arm entries. In the test, GluN2B-YF mice spent less time in the open arms than wild-type (WT) mice (time in open arms: WT, 41.5 ± 3.9%, *n *= 28; YF, 29.0 ± 3.5%, *n *= 31; F_(1,57) _= 5.516, *p *< 0.05, one-way ANOVA) (Figure 1A). We also found that GluN2B-YF mice showed a clear preference for closed arms (percentage of entries into open arms: WT, 50.8 ± 1.8%, *n *= 28; YF, 41.9 ± 2.7%, *n *= 31; F_(1,57) _= 6.908, *p *< 0.05, one-way ANOVA) (Figure 1B). There was no significant difference in the number of total arm entries between GluN2B-YF and WT mice (number of total entries: WT, 44.6 ± 1.9, *n *= 28; YF, 42.6 ± 2.0, *n *= 31; F_(1,57) _= 0.490, *p *> 0.4, one-way ANOVA), suggesting that the locomotor activity of the GluN2B-YF mice was unchanged compared to that of WT mice (Figure 1C). Together with the findings that spontaneous activity of GluN2B-YF mice in the open field test was virtually normal compared to that of the WT mice (data not shown), these results suggest that reduced open arm activity in GluN2B-YF mice is due to increased anxiety rather than motor impairment.

### Increased CRF expression in the amygdala of GluN2B-YF mice

**Figure 2:**
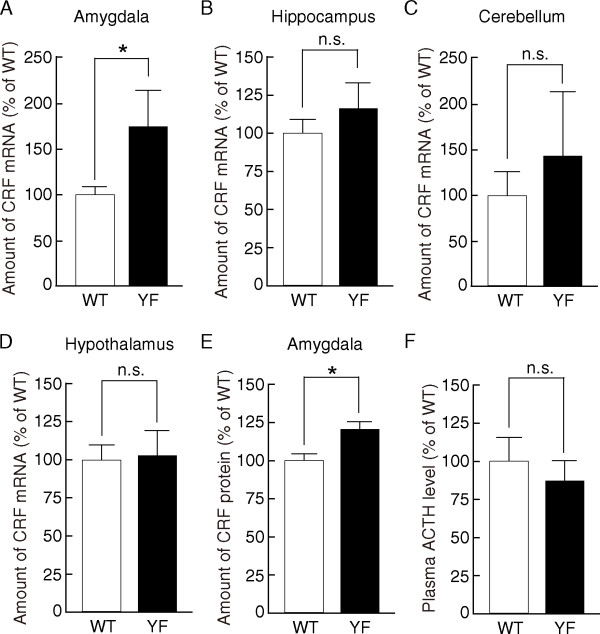
**Increased CRF levels in the amygdala but not in other brain regions in GluN2B-YF mice**. **(A)-(D) **Real-time PCR analyses showed that CRF mRNA levels were increased in the amygdala (WT, *n *= 5; YF, *n *= 5; *p *< 0.05, Student's *t*-test) (A) but not in other brain regions, including the hippocampus (WT, *n *= 5; YF, *n *= 5; *p *> 0.2, Student's *t*-test) (B), cerebellum (WT, *n *= 5; YF, *n *= 5; *p *> 0.2, Student's *t*-test) (C), and hypothalamus (WT, *n *= 5; YF, *n *= 5; *p *> 0.5, Student's *t*-test) (D), of GluN2B-YF mice compared to WT mice. **(E) **Increased expression of CRF in the amygdala was confirmed by ELISA (WT, *n *= 10; YF, *n *= 10; *p *< 0.01, Student's *t*-test). **(F) **The plasma ACTH level of GluN2B-YF mice was not significantly different from that of WT mice (WT, *n *= 9; YF, *n *= 13; *p *> 0.5, Student's *t*-test). The asterisk indicates significant genotype differences. n.s., not significant.

Several neurochemical systems are implicated in anxiety-related behavior in mice [3,22,23]. Among them, we focused on CRF-mediated signaling because many studies have reported that CRF is involved in the fear-potentiated EPM behavior [21]. Relative to wild-type controls, we found, by real-time PCR, that GluN2B-YF mice had markedly increased levels of CRF mRNA in the amygdala, which regulates the behavioral systems involved in the fear response (WT, 100.0 ± 7.3%, *n *= 5; YF, 175.3 ± 41.5%, *n *= 5; *p *< 0.05, Student's *t*-test) (Figure 2A). Interestingly, slight changes in the levels of CRF mRNA in other brain regions, including the hippocampus, cerebellum, and hypothalamus were not significantly different (hippocampus: WT, 100.0 ± 10.3%, *n *= 5; YF, 115.7 ± 19.5%, *n *= 5; *p *> 0.2, Student's *t*-test; cerebellum: WT, 100.0 ± 25.2%, *n *= 5; YF, 145.7 ± 70.1%, *n *= 5; *p *> 0.2, Student's *t*-test; hypothalamus: WT, 100.0 ± 9.7%, *n *= 5; YF, 103.8 ± 16.1%, *n *= 5; *p *> 0.5, Student's *t*-test) (Figure 2B-D). To confirm the increased CRF levels in the amygdala, we performed an ELISA and found that CRF was indeed increased in the amygdalae of GluN2B-YF mice (WT, 100.0 ± 4.4%, *n *= 10; YF, 120.4 ± 5.0%, *n *= 10; *p *< 0.01, Student's *t*-test) (Figure 2E). As expected, there was no significant difference in the CRF levels determined by ELISA in other brain region such as hippocampus between GluN2B-YF and WT mice (CRF levels in the hippocampus: WT, 100.0 ± 6.9, *n *= 10; YF, 105.3 ± 7.0, *n *= 10; *p *> 0.5, Student's *t*-test). In addition, given that CRF plays a key role in hypothalamus-pituitary-adrenal (HPA) axis activation [14], we examined the HPA axis-regulated plasma ACTH levels by ELISA. We found that the plasma ACTH level of GluN2B-YF mice was similar to that of wild-type mice (WT, 419.5 ± 67 pg/ml (100.0 ± 16.0%), *n *= 9; YF, 364.7 pg/ml (86.9 ± 13.2%), *n *= 13; *p *> 0.5, Student's *t*-test) (Figure 2F). This result suggests that the function of HPA axis is normal in GluN2B-YF mice. Thus, it appears that CRF expression is increased in the amygdala but not in other brain regions of GluN2B-YF mice.

### Attenuation of enhanced anxiety-like behavior of GluN2B-YF mice by CRF receptor antagonist

**Figure 3:**
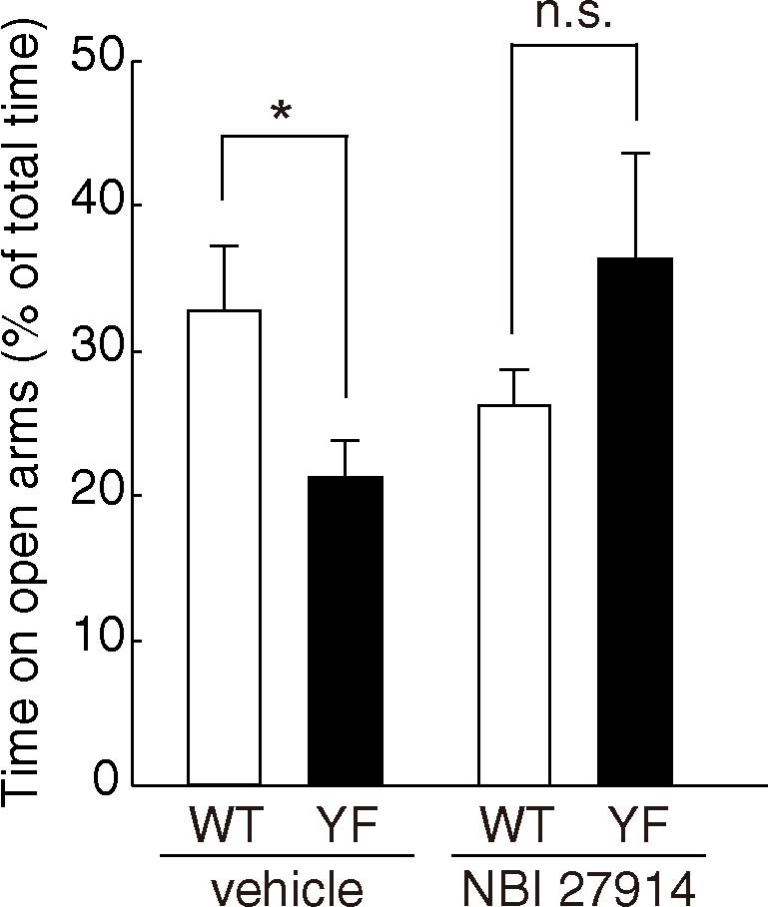
**Abrogation of increased anxiety-related behavior of GluN2B-YF mice by intraperitoneal injection of the CRF_1 _receptor antagonist NBI 27914**. The reduced time spent in the open arms shown by GluN2B-YF mice in the elevated plus-maze test (WT, *n *= 10; YF, *n *= 12; F_(1,37) _= 7.12, *p *< 0.05, two-way ANOVA/Tukey's post-hoc test) was attenuated by intraperitoneal injection of NBI 27914 (WT, *n *= 8; YF, *n *= 9; F_(1,37) _= 2.31, *p *> 0.1, two-way ANOVA/Tukey's post-hoc test). The asterisk indicates significant differences. n.s., not significant.

In the amygdala, CRF modulates anxiety-like behavior by binding CRF_1 _receptor [16]. If the enhanced anxiety-like behavior of GluN2B-YF mice were due to increased CRF expression, blockade of the CRF_1 _receptor might attenuate the enhanced anxiety-like behavior of GluN2B-YF mice. To examine this possibility, we performed the EPM test after injecting mice with the CRF_1 _receptor selective antagonist NBI 27914. There was a significant interaction between genotype and NBI 27914-treatment for the time spent on open arms (F_(1,37) _= 8.30, *p *= 0.0066, two-way ANOVA). Tukey's post-hoc test revealed that vehicle-injected GluN2B-YF mice showed enhanced anxiety-like behavior relative to vehicle-injected WT mice (time on open arms: WT, 32.7 ± 4.5%, *n *= 10; YF, 21.3 ± 2.6%, *n *= 12; F_(1,37) _= 7.12, *p *< 0.05, two-way ANOVA/Tukey's post-hoc test) (Figure 3). In contrast to the vehicle-injection, we found that the enhanced anxiety-like behavior exhibited by GluN2B-YF mice was attenuated by acute intraperitoneal injection of NBI 27914 (time on open arms: WT, 25.8 ± 2.6%, *n *= 8; YF, 36.5 ± 7.4%, *n *= 9; F_(1,37) _= 2.31, *p *> 0.1, two-way ANOVA/Tukey's post-hoc test) (Figure 3). These results argue that activated CRF_1 _receptor-mediated signaling causes the enhanced anxiety-like behavior of GluN2B-YF mice. In addition, in contrast to the case seen in Figure 1, slight decrease in the number of entries into open arms of vehicle-injected GluN2B-YF mice was not significantly different (vehicle-injected WT, 48.5 ± 3.6%, *n *= 10; vehicle-injected YF, 40.1 ± 2.5%, *n *= 12; *p *= 0.08, two-way ANOVA/Tukey's post-hoc test; NBI 27914-injected WT, 40.4 ± 5.0%, *n *= 8; NBI 27914-injected YF, 42.8 ± 4.9%, *n *= 9; *p *= 0.68, two-way ANOVA/Tukey's post-hoc test), probably because of injection stress [19].

### De-phosphorylation of Tyr-1472 and increased CRF expression in the amygdala of wild-type mice after the EPM test

**Figure 4:**
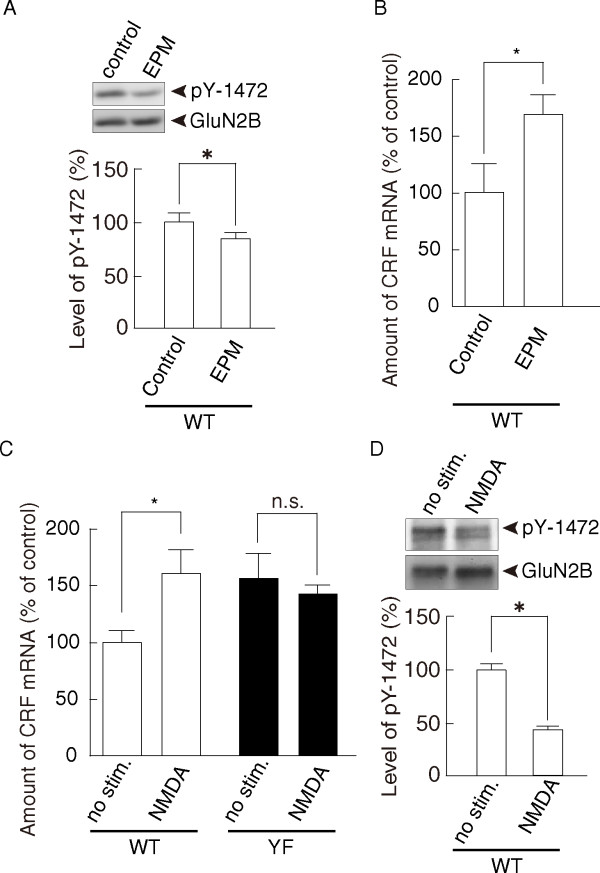
**De-phosphorylation of Tyr-1472 and up-regulation of CRF expression after the EPM test and by NMDA-receptor stimulation**. **(A) **The level of Tyr-1472 phosphorylation in the amygdala of WT mice was decreased by the EPM test (control, *n *= 6; EPM test, *n *= 4; *p *< 0.05, Student's *t*-test). A representative blot is shown in the upper panel. **(B) **Real-time PCR analyses showed that CRF expression in the amygdala of WT mice was increased after the EPM test (control, *n *= 6; EPM test, *n *= 4; *p *< 0.05, Student's *t*-test). **(C) **Real-time PCR analyses showed that NMDA stimulation (100 μM NMDA for 7 min) increased CRF expression in the amygdalae of WT mice (control, *n *= 6; NMDA stimulation, *n *= 6; *p *< 0.05, Student's *t*-test) but not in GluN2B-YF mice (control, *n *= 6; NMDA stimulation, *n *= 6; *p *> 0.2, Student's *t*-test). **(D) **The same NMDA stimulation also induced de-phosphorylation of Tyr-1472 in the amygdalae of WT mice (control, *n *= 5; NMDA stimulation, *n *= 5; *p *< 0.05, Student's *t*-test). A representative blot is shown in the upper panel. The asterisk indicates significant genotype differences. n.s., not significant.

We next investigated whether the level of Tyr-1472 phosphorylation in the amygdala of wild-type mice was affected by the EPM test. At 10 min after the test, the amygdala was resected, and the total lysates and RNAs were prepared. As shown in Figure 4A, the level of Tyr-1472 phosphorylation was significantly decreased in the amygdala of wild-type mice exposed to the EPM test compared with control wild-type mice (control: 100.0 ± 8.5%, *n *= 6; EPM test: 85.1 ± 5.3%, *n *= 4; *p *< 0.05, Student's *t*-test). Interestingly, the level of CRF mRNA in the amygdala was increased by the EPM test (control: 100.0 ± 24.3%, *n *= 6; EPM test: 168.7 ± 17.3%, *n *= 4; *p *< 0.05, Student's *t*-test) (Figure 4B). These data further support that Tyr-1472 phosphorylation is relevant to the anxiety-like behavior and negative regulation of CRF expression in the amygdala.

### Induction of de-phosphorylation of Tyr-1472 and increased CRF expression by NMDA receptor stimulation

We then examined whether NMDA receptor activity is involved in the regulation of CRF expression in the amygdala. Coronal sections including the amygdala were stimulated with 100 μM NMDA for 7 min, followed by a wash-out period of 15 min. Then, the amygdala was rapidly dissected out and RNA was isolated to examine the level of CRF mRNA expression (Figure 4C). We found that NMDA receptor stimulation increased CRF mRNA expression in wild-type slices (control: 100.0 ± 10.5%, *n *= 6; NMDA stimulation: 160.1 ± 20.3%, *n *= 6; *p *< 0.05, Student's *t*-test). Interestingly, the same stimulation also induced de-phosphorylation of GluN2B Tyr-1472 (the level of Tyr-1472 phosphorylation: control, 100.0 ± 5.8%, *n *= 5; NMDA stimulation, 42.5 ± 3.1%, *n *= 5; *p *< 0.05, Student's *t*-test) (Figure 4D). In contrast to wild-type slices, CRF mRNA expression levels in the slices from GluN2B-YF mice were virtually unchanged by the same NMDA stimulation (control: 153.3 ± 20.5%, *n *= 6; NMDA stimulation: 143.1 ± 7.5%, *n *= 6; *p *> 0.2, Student's *t*-test) (Figure 4C). Thus, NMDA receptor stimulation is a likely trigger for increased CRF mRNA expression through de-phosphorylation of Tyr-1472 in the amygdala.

## Discussion

In this study, we showed that Tyr-1472 phosphorylation of GluN2B is a negative regulator of CRF mRNA expression in the amygdala. Behaviorally, deficient Tyr-1472 phosphorylation leads to enhanced anxiety-like behavior, which is consistent with enhanced CRF signaling [14,17,18]. We further demonstrated that acute intraperitoneal injection of NBI 27914, a selective CRF_1 _receptor antagonist, attenuated the anxiety-like behavior of GluN2B-YF mice. Given the established role of CRF in anxiety [14,16,17], it is likely that the enhanced anxiety phenotype observed in GluN2B-YF mice is linked to increased CRF expression in the amygdala.

Considering that Tyr-1472 phosphorylation is required for the NMDA receptor-mediated signaling [13], the present finding that GluN2B-YF mice exhibit increased anxiety-like behavior is consistent with previous pharmacological and genetic studies in rodents showing the anxiolytic-like effects of NMDA receptor blockade [24,25]. Strikingly, intra-amygdala injection of an NMDA receptor antagonist, MK-801, prevents stress-induced increases in anxiety-like behavior in the EPM test [25], suggesting that NMDA receptors, especially in the amygdala, play a key role in regulating anxiety-like behavior. Interestingly, we found that the level of CRF was markedly increased in the amygdala of GluN2B-YF mice but not in other brain regions such as the hippocampus, cerebellum, and hypothalamus (Figure 2B-D). Thus, the Tyr-1472 phosphorylation-dependent regulation of NMDA receptors in the amygdala is likely to be responsible for anxiety-like behavior. Tissue specific conditional GluN2B-YF mice would be useful for analyzing the differential roles of Tyr-1472 phosphorylation.

CRF in the amygdala contributes to anxiety because injection of CRF antagonists or CRF_1 _receptor antisense oligonucleotides into the amygdala reduces anxiety-like behavior in rats [19,20]. In amygdala-derived neuronal cultures, NMDA receptor stimulation induces CRF release [26], suggesting that a functional NMDA receptor system regulates CRF signaling in the amygdala. In this study, we found that the YF mutation in the Tyr-1472 phosphorylation site leads to increased CRF mRNA expression (Figure 2). Correlating with this, NMDA receptor stimulation induced tyrosine de-phosphorylation of Tyr-1472 and up-regulation of CRF mRNA expression in amygdala slices (Figure 4). Thus, Tyr-1472 phosphorylation links NMDA receptor activity and CRF expression in the amygdala: however, the mechanisms underlying enhanced CRF mRNA expression in GluN2B-YF mice remain to be determined. We previously found that Tyr-1472 phosphorylation regulates NMDA receptor-mediated CaMKII signaling in the amygdala [13]. A simple model would predict that downstream NMDA receptor-mediated CaMKII signaling regulates CRF mRNA expression in the amygdala.

One major neuroendocrine system underlying an individual's capacity to cope with stress is the HPA axis [14]. Besides the regulation of anxiety-like behavior, CRF is a key coordinator of the HPA axis and an essential component in the mediation of behavioral responses to stress [14]. In contrast to the increased CRF levels in the amygdala (Figure 2A), we did not find any significant differences in hypothalamic CRF levels between GluN2B-YF mice and WT mice (Figure 2D). Consistent with these findings, the basal levels of plasma ACTH in GluN2B-YF mice were virtually unchanged compared to those of WT mice (Figure 2F). Thus, the function of Tyr-1472 phosphorylation may be different between brain regions. Alternatively, in the hypothalamus, some compensatory event might have occurred to mask the hypothalamic phenotypes in GluN2B-YF mice.

There is widespread interest in CRF_1 _receptor antagonists for the treatment of anxiety disorders [21,27]: however, these potential therapies can influence the function of the HPA axis in response to stress [28]. Tyr-1472 phosphorylation in GluN2B regulates anxiety-like behavior through regulation of amygdaloid CRF expression without altering the function of the HPA axis. Therefore, blocking Tyr-1472 phosphorylation in the amygdala may be a clinically effective means of treating anxiety disorders without the potential risks associated with blocking of the function of the HPA axis.

## Conclusions

In summary, the present study demonstrates that the cellular interaction between CRF signaling and NMDA receptors, especially Tyr-1472 phosphorylation of GluN2B, is necessary for the regulation of anxiety-like behavior. GluN2B-YF mice should serve as a useful animal model to study the pathogenesis of anxiety disorders and to develop therapeutic drugs for the disease.

## Methods

### Animals

Heterozygous GluN2B-YF mice [13] were successively backcrossed to C57BL/6J mice to yield subsequent generations with a pure C57BL/6J genetic background. F10 heterozygous mice were crossed to each other to yield homozygous mice and wild-type littermates. Male 8-12-week old mice were used in this study. All experiments and analyses were done in a completely blind manner. Experiments with animals were carried out in accordance with the guidelines for animal use issued by the Committee of Animal Experiments, Institute of Medical Science, University of Tokyo.

### Elevated plus-maze (EPM) test

The elevated plus-maze test (EP-3002; O' Hara & Co., Ltd., Tokyo, Japan) consisted of two open arms (25 × 5 cm) and two enclosed arms of the same size extending from a central area (5 × 5 cm) and elevated 50 cm from the ground. The ambient light level was 30 lux. The mice were placed in the central square of the maze facing one of the open arms. Mouse behavior was recorded during a 10 min test period with a Macintosh computer using Image EP 2.13× and Image EPC 2.03s× (O'Hara & Co., Ltd.), a modified software based on the public domain of NIH Image program. The following conventional parameters were recorded: the number of entries into open or closed arms and the time spent in open or closed arms.

### Real-time PCR

Mice were anesthetized with halothane and immediately decapitated. Brains were then rapidly removed and frozen by liquid nitrogen. Serial coronal sections (400 μm thickness) were cryosectioned, and the various brain regions were collected with a biopsy puncher. Total RNA was isolated from the brain regions and reverse transcribed with Superscript III (Invitrogen, Carlsbad, CA, USA). Real-time PCR was performed with TaqMan primers on an ABI PRISM 7900HT system (Applied Biosystems, Foster City, CA, USA) according to the supplier's protocol. The following intron-spanning primer sets were used: HPRT (internal control), Mm0046968_m1; CRF, Mm01293920_S1 (Applied Biosystems). Relative expression levels were determined according to the 2-ΔΔCt method (Applied Biosystems, User Bulletin).

### Measurements of CRF protein level

Serial coronal sections (200 μm) were cryosectioned, and the amygdalae were collected [29]. CRF protein levels were measured by using an enzyme-linked immunoassay kit (Mouse/Rat CRF-HS ELISA kit (YK131), Yanaihara Institute Inc., Shizuoka, Japan) according to the supplier's protocol.

### Measurements of plasma adrenocorticotropin-releasing hormone (ACTH) levels

Mice were killed by rapid decapitation, and blood was collected at around 9:00 AM. Serum was isolated and frozen at -80°C until analysis. Basal plasma ACTH levels were measured using an enzyme-linked immunoassay (SRL Inc., Tokyo, Japan).

### Intraperitoneal injection of CRF_1 _receptor antagonist

A non-peptide selective CRF1 receptor antagonist, NBI 27914 (dissolved at 10 mg/ml in DMSO, 10 mg/kg body weight, Tocris, Bristol, UK) [30] or vehicle (DMSO) was injected intraperitoneally 45 min before the EPM test.

### Pharmacological treatment of brain slices

Coronal slices including the amygdala (400 μm thickness) were prepared from 8- to 10-week-old mice and placed in an interface-type holding chamber for at least 3 h. Slices were preincubated in ACSF [13] for 1 h and then stimulated with 100 μM NMDA for 7 min.

### Preparation of lysate, immunoprecipitation and immunoblotting

Preparation of lysate from the mouse brain and immunoblotting were performed as described previously [13]. For quantification, the immunoreacted protein bands were analyzed with the NIH image software. The rabbit polyclonal antibody against phospho-Tyr-1472/GluN2B was described previously [31]. The mouse monoclonal antibody against GluN2B was purchased from Millipore (Billerica, MA, USA).

### Statistical analysis

All data are expressed as the means **± **SEM. Statistical analysis was performed using Student's *t *test, one-way ANOVA, two-way ANOVA, and Tukey's post hoc test. Differences with *p *< 0.05 were considered as significant.

## Abbreviations

ACTH: adrenocorticotropin-releasing hormone; ANOVA: analysis of variance; CRF: corticotropin-releasing factor; EPM: elevated plus-maze; HPA: hypothalamic-pituitary-adrenocortical; NMDA: *N*-methyl-D-aspartate;

## Competing interests

The authors declare that they have no competing interests.

## Authors' contributions

MD, TT, YK, TM, TY, and TN designed the project. MD, TT, YK, KY, TI, SH, RH, HU, and TN performed experiments and analyzed the data. TM, TY, and TN wrote the manuscript and supervised the project. All authors read and approved the final manuscript.
